# α-Cyclodextrin/Moringin Induces an Antioxidant Transcriptional Response Activating Nrf2 in Differentiated NSC-34 Motor Neurons

**DOI:** 10.3390/antiox13070813

**Published:** 2024-07-06

**Authors:** Agnese Gugliandolo, Gabriella Calì, Claudia Muscarà, Osvaldo Artimagnella, Patrick Rollin, Daniele Perenzoni, Renato Iori, Emanuela Mazzon, Luigi Chiricosta

**Affiliations:** 1 IRCCS Centro Neurolesi “Bonino-Pulejo”, Via Provinciale Palermo, Contrada Casazza, 98124 Messina, Italy; 2 Institute of Organic and Analytical Chemistry (ICOA), Université d’Orléans, UMR 7311, BP 6759, F-45067 Orléans, Cedex 2, France; 3 Department of Food Quality and Nutrition, Research and Innovation Centre, Fondazione Edmund Mach (FEM), Via E. Mach 1, 38098 San Michele all’Adige, Italy

**Keywords:** Moringin, motor neurons, isothiocyanate, Nrf2, transcriptomic analysis

## Abstract

Oxidative stress is a common feature of neurodegenerative diseases. Different natural compounds mediate neuroprotective effects by activating the Nrf2 antioxidant response. Some isothiocyanates are Nrf2 activators, including Moringin (MOR). In this study, the transcriptional profile of differentiated NSC-34 motor neurons was evaluated after treatment for 48 h and 96 h with concentrations of 0.5, 5, and 10 µM of a new MOR formulation obtained with α-cyclodextrin (α-CD). All the concentrations increased gene expression and cytoplasmic protein levels of Nrf2 at 96 h. However, the highest dose also increased nuclear Nrf2 levels at 96 h. Then, Nrf2 interactors were selected using STRING, and common biological process (BP) terms between the groups were evaluated. α-CD/MOR was able to modulate BP related to responses to oxidative stress, proteostasis, and autophagy. Specifically, the treatment with 10 µM of α-CD/MOR for 96 h induced genes involved in glutathione synthesis and proteasome subunits and reduced the expression of genes related to endoplasmic reticulum stress. Moreover, this group showed the lowest levels of the apoptotic markers Bax, cleaved caspase 9, and cleaved caspase 3. These results indicate the beneficial effects of prolonged α-CD/MOR supplementation that are mediated, at least in part, by Nrf2 activation. Then, α-CD/MOR could be a valuable treatment against neurodegenerative diseases, in particular motor neuron degeneration.

## 1. Introduction

### 1.1. Cruciferous Vegetables and Their Phytochemicals

Crucifer vegetables, belonging to the order *Brassicaceae* and *Moringaceae*, have been reported to show health-promoting effects in the treatment and prevention of some diseases. Some of the cruciferous vegetables commonly consumed as food include cabbage, cauliflower, broccoli, kale, and wasabi [1]. Specifically, *Moringa oleifera*, also known as “horseradish tree” or “drumstick tree”, has been consumed for both nutritional and medicinal purposes [2].

The phytochemicals responsible for their protective effects include minerals, compounds with phenolic groups, vitamins, fatty acids, nitrites, thiocyanates, glucosinolates (GLs), and their hydrolytic products, namely isothiocyanates (ITCs) [3]. Specifically, GLs and ITCs have been largely studied for their properties. GLs are mainly present in the seeds, roots, and leaves of crucifer vegetables, which also contain myrosinase in separate cell compartments. When they come into contact, myrosinase causes the hydrolysis of GLs, forming ITCs. Different GLs yield different ITCs after hydrolysis: glucoraphanin produces sulforaphane (SFN), while glucomoringin (GMG) yields moringin (MOR). ITCs exert anti-inflammatory, antioxidant, anti-cancer, neuroprotective, and cardioprotective effects [4,5,6]. Specifically, MOR has shown anti-microbial [7], antioxidant [8,9], anti-inflammatory [10], anti-cancer [11], and neuroprotective effects [12,13,14] in both in vitro and in vivo models. 

### 1.2. Oxidative Stress and Its Role in Motor Neuron Degeneration

The antioxidant effect of ITCs is particularly important and is associated with their neuroprotective capacity. Indeed, neurodegenerative diseases are characterized by excessive free radical generation within neurons, leading to neuronal cell death. In particular, motor neurons are sensitive to oxidative stress. Oxidative stress markers were found in patients and models with amyotrophic lateral sclerosis (ALS), a rapidly progressive neurodegenerative disease characterized by the gradual loss of upper and lower motor neurons. Moreover, genes associated with ALS are involved in oxidative stress, such as *SOD1*, *TARDBP*, *FUS,* and *C9ORF72* [15]. Oxidative stress negatively impacts cellular function due to the oxidation of lipids, proteins, and nucleic acids [16]. The increase in oxidative stress in ALS is associated with mitochondrial dysfunction, a decrease in ATP and glutathione (GSH) levels, impaired autophagy, and proteasomal degradation, leading to endoplasmic reticulum (ER) stress. Finally, all these alterations lead to motor neuron cell death and degeneration [17]. 

### 1.3. The Antioxidant Defense System and the Role of MOR

The antioxidant defense system includes scavenging and repairing molecules, including enzymes and molecules such as GSH that are involved in maintaining redox balance [18]. Among the main molecules involved in cellular responses against oxidative stress, there is nuclear factor erythroid 2-related factor 2 (Nrf2). Nrf2 is a key redox-sensitive transcriptional factor that regulates the expression of many antioxidants and cytoprotective genes and phase II enzymes and is negatively regulated by Kelch-like ECH-associated protein (Keap1), an adaptor protein able to bind Nrf2 and induce its polyubiquitination and subsequent proteasomal degradation in the cytosol. Stress conditions or Nrf2 inducers can lead to the dissociation of the Nrf2/Keap1 complex through Keap1 degradation, allowing Nrf2 translocation into the nucleus. At the nuclear level, Nrf2 binds to the antioxidant response element (ARE) to induce the expression of genes encoding for enzymes involved in detoxification, stress response, and proteostasis [19,20]. Different ITCs are well-known activators of Nrf2, such as SFN [21]. However, MOR has also been shown to be able to activate Nrf2 [13,22]. Interestingly, antioxidant therapies were proposed as treatments for ALS, including Nrf2 inducers [15]. 

In this study, we evaluated the effects of prolonged treatment of differentiated NSC-34 motor neuronal cells with a new formulation of MOR obtained by combining MOR with α-cyclodextrin (α-CD). Indeed, MOR is poorly soluble and unstable in an aqueous medium. For this reason, the new formulation α-CD/MOR was developed to improve solubility and stability in an aqueous solution [23]. Specifically, we treated differentiated NSC-34 cells with different concentrations of α-CD/MOR (0.5, 5, and 10 µM) for 48 h and 96 h to evaluate the effects of a prolonged treatment using next generation sequencing (NGS) analysis. 

## 2. Materials and Methods

### 2.1. Preparation of the α-CD/MOR Complex 

MOR was synthesized by hydrolysis catalyzed by myrosinase of GMG, extracted from *Moringa oleifera* seeds (cake powder PKM2 provided by Indena India Pvt. Ltd., Bangalore, India), and purified using reverse-phase chromatography. The protocol for its preparation is reported in [24,25,26]. The α-CD/MOR complex was prepared as reported in [12] and characterized as described in Mathiron et al. [23].

### 2.2. NSC-34 Culture, Differentiation, and Treatment 

The NSC-34 cell line was purchased from Cedarlane Corporation (Burlington, ON, Canada). The maintenance medium is composed as follows: DMEM High Glucose supplemented with 10% Fetal Bovine Serum (FBS), 1% penicillin/streptomycin, and 1% L-Glutamine (Sigma-Aldrich, Merck KGaA, Darmstadt, Germany). NSC-34 cells were seeded in 6-well plates. With the aim of inducing cell differentiation, 24 h after seeding, NSC-34 cells were incubated for 5 days with the following differentiation medium: 1:1 DMEM/F-12 (Ham), 1% FBS, 1% L-glutamine, 0.5% penicillin/streptomycin, and 1 μM retinoic acid (Sigma-Aldrich, Saint Louis, MO, USA). 

At the end of differentiation, cells were treated with different concentrations of α-CD/MOR, namely 0.5 µM, 5 µM, and 10 µM, for 48 and 96 h. α-CD/MOR was dissolved directly in the medium, and fresh medium containing fresh α-CD/MOR was changed every 24 h until the end of the treatment. 

### 2.3. Cell Viability

With the aim of evaluating cell viability, NSC-34 cells were cultured in 96-well plates, differentiated, and treated with α-CD/MOR as reported in paragraph 2.2, and MTT assay was performed. After 48 h and 96 h treatments, the medium was replaced with fresh medium containing Thiazol Blue Tetrazolinium Bromide (MTT) at a concentration of 0.5 mg/mL (Sigma-Aldrich Merck KGaA, Darmstadt, Germany), and the plates were incubated for 4 h at 37 °C. After 4 h, the crystals were dissolved in acidic isopropanol. The microplate reader BioTek Synergy H1 (Agilent, Santa Clara, CA, USA) was used to measure the optic density.

### 2.4. RNA Extraction and Library Preparation 

NSC-34 cells were collected at the end of the 48- and 96 hour treatments with α-CD/MOR, and total RNA was obtained with the Maxwell^®^ RSC Simply RNA Cells Kit (Promega, Madison, WI, USA). A cDNA library for transcriptomic analysis was prepared using the TruSeq RNA Exome (Illumina, San Diego, CA, USA) following the protocol. 

### 2.5. Comparative Transcriptomic and Linear Model Prediction 

The quality score of each base of the runs was checked using FastQC (version 0.11.9, Babraham Institute, Cambridge, UK). Trimmomatic (version 0.40-rc1, Usadel Lab, Aachen, Germany) [27] was used to remove the adapters from the sequences. The reads were then mapped against the genome of Mus Musculus (version vM28) from GENCODE using the STAR RNA-seq aligner (version 2.7.10a_alpha_220207, New York, NY, USA) [28]. 

The counting of reads was computed with HTSeq (version 0.13.5) [29]. Then, all the analysis were made in R version 4.2.0 (R Core Team). The fold change extracted by the comparative analysis was obtained through the DESeq2 library version 1.36.0 [30]. The associated *p*-values were corrected and defined as q-values using the Benjamini-Hochberg method with a threshold of 0.05 to remove false-positive genes. A gene with a q-value lower than 0.05 was defined as a Differentially Expressed Gene (DEG). The DEGs were associated with the ontology term using BiomaRt version 2.58.2 based on the Mus Musculus database (org.Mm.eg.db) version 3.18.0. For each comparison, the heatmaps were computed using the pheatmap library version 1.0.12. The value of the cells was computed in two different ways. The first one is a ratio of the number of DEGs included in the term to the number of genes included in the term. The second one is a normalized score obtained by multiplying the first value by the sum of the absolute fold change of DEGs included in the term itself. To dynamically obtain the number of clusters for the terms in each comparison, a generalized linear model with a gaussian family and logarithmic link was used. The model was used to fit the height of the dendrogram obtained in the comparison. Then, the predicted value was compared against the original value. The first positive value obtained from the difference between the actual and predicted heights was used to choose the number of clusters in the comparison. Finally, the network of gene ontology (GO) terms was made using iGraph (version 2.0.3). The data manipulation was performed using dplyr version 1.1.4 and stringr version 1.5.1. 

### 2.6. Nrf2 Network and Gene Ontology Analysis 

All the interactors of the Nrf2 protein were then retrieved. In particular, the protein Nrf2 is encoded in mouse by the nuclear factor erythroid-derived 2 (*Nfe2l2*) gene. Thus, the interactors were retrieved using the STRING database (accessed on 28 February 2024) [31]. All default parameters were retained. All the interactors were obtained. 

Moreover, the overrepresentation terms of biological process (BP) terms of GO were obtained using the Panther database (accessed on 28 February 2024) [32]. We used a slim database that is manual-curated.

### 2.7. Protein Extraction and Western Blot 

NSC-34 cells were harvested at the end of the 48- and 96 hour treatments, and proteins were obtained using the NE-PER™ Nuclear and Cytoplasmic Extraction Reagents (Thermo Scientific™, Waltham, MA, USA), following the manufacturer’s instructions. Protein concentration was evaluated with Bradford assay (Bio-Rad, Hercules, CA, USA). Thirty micrograms of proteins were heated for 5 min at 95 °C. Then, proteins were loaded and resolved by SDS-polyacrylamide gel electrophoresis (SDS-PAGE). At the end, they were transferred onto a PVDF membrane (Immobilon-P, Millipore, Burlington, MA, USA). 5% skim milk dissolved in TBS was used to block membranes for 1 h at room temperature. Then, membranes were incubated overnight at 4 °C with the following antibodies: anti-p62 (1:1000; Cell Signaling Technology, Danvers, MA, USA); anti-caspase 3 (1:1000; Cell Signaling Technology, Danvers, MA, USA); anti-cleaved caspase 3 (1:1000; Cell Signaling Technology, Danvers, MA, USA); anti-Nrf2 (1:500; Santa Cruz Biotechnology, Dallas, TX, USA); anti-Bax (1:1000; Cell Signaling Technology, Danvers, MA, USA); anti-LC3 (1:1000; Cell Signaling Technology, Danvers, MA, USA); anti-Beclin 1 (1:1000; Cell Signaling Technology, Danvers, MA, USA); anti-cleaved caspase 9 (1:250; Abcam, Cambridge, UK); anti-caspase 9 (1:1000; Abcam, Cambridge, UK); After washing with TBS 1×, membranes were incubated with HRP-conjugated anti-rabbit antibody (1:2000 or 1:1000 for cleaved caspase 9; Santa Cruz Biotechnology Inc., Dallas, TX, USA) for 1 h at room temperature. The protein bands were visualized using the Immobilion Forte Western HRP Substrate (Millipore Corporation, Burlington, MA, USA). Protein band acquisition was carried out using the ChemiDoc™ XRS + System (Bio-Rad, Hercules, CA, USA). Protein band quantification was carried out using ImageJ 1.53t software. Membranes were stripped with Restore Western Blot buffer (Thermo Scientific, Meridian, Rockford, IL, USA) and then incubated with GAPDH HRP-conjugated antibody (1:1000; Cell Signaling Technology, Danvers, MA, USA) for cytoplasmic extracts or with Lamin B1 (1:500; Cell Signaling Technology, Danvers, MA, USA) for nuclear extracts, used as loading controls. The expressions of cleaved caspases 3 and 9 were normalized on the expressions of caspases 3 and 9, respectively. LC3-II expression was normalized by the expression of LC3-I. The original uncropped blots are available in Figures S1–S7.

### 2.8. Statistical Analysis 

Cell viability and western blot statistical analysis were performed with GraphPad Prism version 10.2.3 software (GraphPad Software, La Jolla, CA, USA). Multiple comparisons among the groups were performed using the one-way ANOVA test and the Bonferroni post hoc test. A *p*-value ≤ 0.05 was considered statistically significant. The results are represented as the mean ± standard deviation (SD).

## 3. Results

### 3.1. Cell Viability

**Figure 1:**
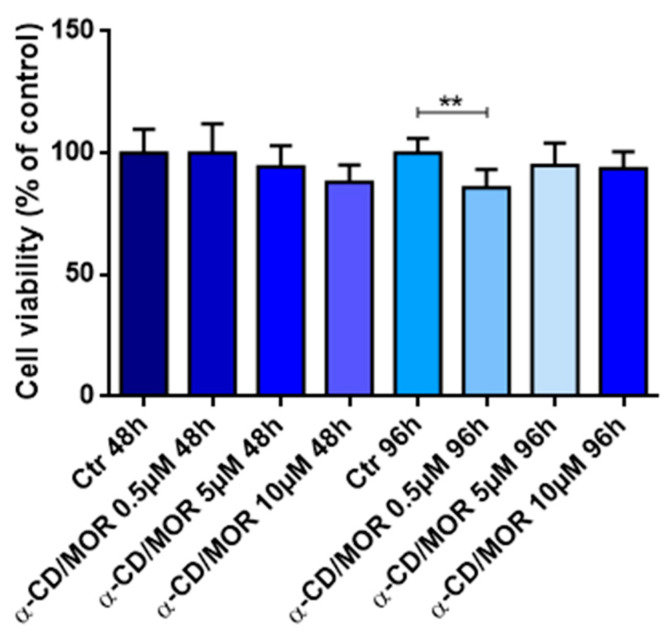
Cell viability of differentiated NSC-34 cells treated for 48 h and 96 h with 0.5, 5, and 10 µM α-CD/MOR. A slight decrease in cell viability was found only after 96 h at a concentration of 0.5 µM α-CD/MOR. ** *p* < 0.01.

Differentiated NSC-34 cells were treated for 48 h and 96 h with different α-CD/MOR doses, namely 0.5, 5 and 10 µM, and cell viability was evaluated. Only after 96 h a slight decrease in cell viability was found at the concentration of 0.5 µM α-CD/MOR, as shown in Figure 1.

### 3.2. Transcriptomic Comparative Analysis 

At the end of the treatments, NSC-34 cells were collected, and transcriptomic analysis was carried out. The comparative analysis was made for each dose of α-CD/MOR at 0.5, 5, and 10 µM. Thus, the groups treated with each dose of α-CD/MOR for 48 h were compared against CTR at 48 h. Additionally, the groups treated with each dose of α-CD/MOR for 96 h were compared against CTR at 96 h. In detail, at 48 h, 5546 DEGs were found in the comparison CTR vs. 0.5 µM α-CD/MOR (2665 upregulated, 2881 downregulated, Table S1), 5796 DEGs were found in the comparison CTR vs. 5 µM α-CD/MOR (2871 upregulated, 2925 downregulated, Table S2), and 6081 DEGs were found in the comparison CTR vs. 10 µM α-CD/MOR (2979 upregulated, 3102 downregulated, Table S3). On the other hand, at 96 h, 5005 DEGs were found in the comparison CTR vs. 0.5 µM α-CD/MOR (2524 upregulated, 2481 downregulated, Table S4), and 5320 DEGs were found in the comparison CTR vs. 5 µM α-CD/MOR (2611 upregulated, 2709 downregulated, Table S5), 5964 DEGs were found in the comparison CTR vs. 10 µM α-CD/MOR (2957 upregulated, 3007 downregulated, Table S6).

### 3.3. Nrf2 Gene and Protein Expression Analysis 

It is well known that ITCs, thanks to the –N=C=S group, are powerful activators of Nrf2 [21]. Also, α-CD/MOR showed the ability to activate Nrf2 in different models. Then, at first, we evaluated the expression of the gene *Nfe2l2* encoding for Nrf2. The analysis evidenced that *Nfe2l2* was significantly upregulated at 48 h only with only the concentration of 10 µM α-CD/MOR, while after 96 h all the concentrations were able to significantly upregulate its expression. Western blot analysis was carried out to evaluate cytoplasmic and nuclear Nrf2 protein levels. 

**Figure 2:**
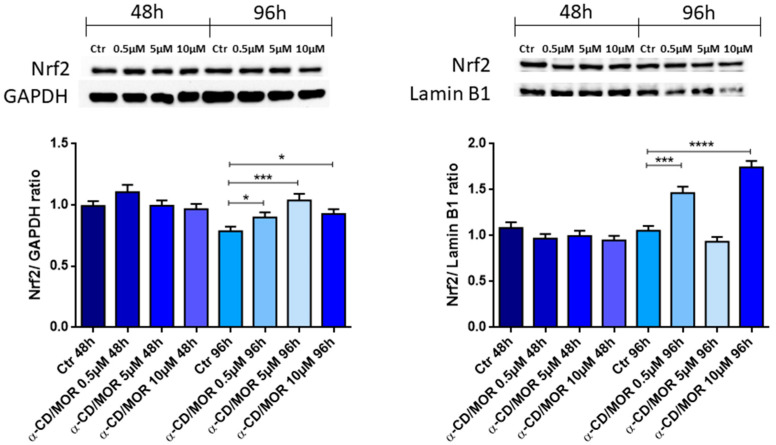
Western blot analysis of Nrf2 cytoplasmic and nuclear levels. Cytoplasmic Nrf2 protein levels increased at all the concentrations tested at 96 h. The concentration of 10 μM α-CD/MOR induced the highest increase in Nrf2 nuclear levels in differentiated NSC-34 cells after 96 h. * *p* < 0.05; *** *p* < 0.001; **** *p* < 0.0001.

Western blot analysis on the cytoplasmic fraction evidenced the increase in Nrf2 protein levels at 96 h, with all the concentrations tested, as shown in Figure 2 (left panel). Western blot analysis also revealed the ability of α-CD/MOR to induce Nrf2 activation and, consequently, its nuclear translocation. Specifically, western blot analysis showed an increase in nuclear levels of Nrf2 at 96 h with concentrations of 0.5 and 10 μM α-CD/MOR. As shown in Figure 2 (right panel), the statistical analysis highlighted the highest nuclear levels of Nrf2 with 10 μM α-CD/MOR at 96 h.

### 3.4. Nrf2 Interactor Selection and Gene Ontology (GO) Analysis 

Given these results, to put a focus on the effect of α-CD/MOR associated with Nrf2, we extracted all DEGs that are interactors with Nrf2. Specifically, the inspection of the network of Nrf2 was made with STRING, and it revealed 426 nodes and 20,956 edges (Table S7).

Thus, we kept, for each comparison, only DEGs that are also interactors of Nrf2. In detail, at 48 h, 145 interactors were found at 0.5 µM α-CD/MOR, 141 interactors were found at 5 µM α-CD/MOR, and 160 were found at 10 µM α-CD/MOR. On the other hand, at 96 h, 129 interactors were found at 0.5 µM α-CD/MOR, 142 interactors were found at 5 µM α-CD/MOR, and 167 interactors were found at 10 µM α-CD/MOR. 

Then, we performed GO overrepresentation analysis related to slim BP terms for each comparison (Tables S8–S13). To observe, which terms have a huge impact, we retrieved all genes that in the GO database are associated with all the found terms. To observe the differences in the effects obtained using α-CD/MOR at different doses and different time points, we performed different analyses. In the first, we kept all terms common to each comparison to observe the effects of α-CD/MOR that are independent from timing and doses. In the second analysis, we kept all terms common at 0.5, 5, and 10 μM for 48 h to see the time-dependent effect, and we completed the same analysis for the groups at 96 h. All comparisons showed 54 common terms (Table S14). All doses showed 34 common terms at 48 h (Table S15) and 3 common terms at 96 h (Table S16). 

**Figure 3:**
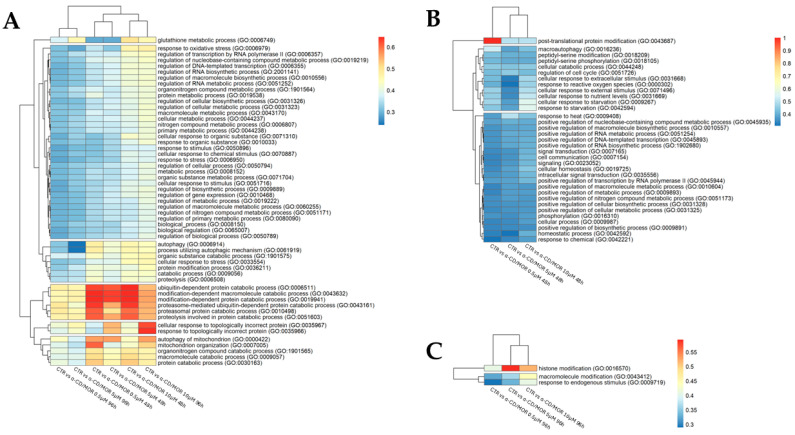
Heatmap of biological processes commonly overrepresented in the different comparisons based on gene ratio. Frame (**A**) is related to the ontologies commonly overrepresented in all the comparisons. The frames (**B**,**C**) show the ontologies commonly overrepresented in all doses only at 48 h or 96 h, respectively. The cell filling represents the ratio between the number of DEGs observed in a comparison included in a specific GO and the number of genes included in that ontology term. Red represents groups with a higher ratio, while blue represents those with lower ratio values. The dendrogram clusters are obtained using a generalized linear model.

Nevertheless, to observe more scenarios in our analysis, we used different scores. The first one, shown in Figure 3, is related to the ratio of the total number of DEGs observed in a specific term for a specific group over the total number of genes that are included in that term. This score highlights quantitatively the terms that are more impacted. The clusters highlight BP with a similar trend in the groups. Interestingly, the BP “response to oxidative stress” is common to all the groups, but the concentration of 10 µM α-CD/MOR at both time points showed a higher ratio. The same is true for the BP “glutathione metabolic processes “and for the BP related to nucleic acid metabolism. The other BPs that showed a high ratio are related to autophagy, protein catabolism, topologically incorrect proteins, and mitochondrial organization. Looking at the heatmaps regarding the separate timepoints, only the BP “post-translational protein modification” and “histone modification” showed higher ratio for 48 and 96 h, respectively. The other BP presented a similar ratio in all the groups.

**Figure 4:**
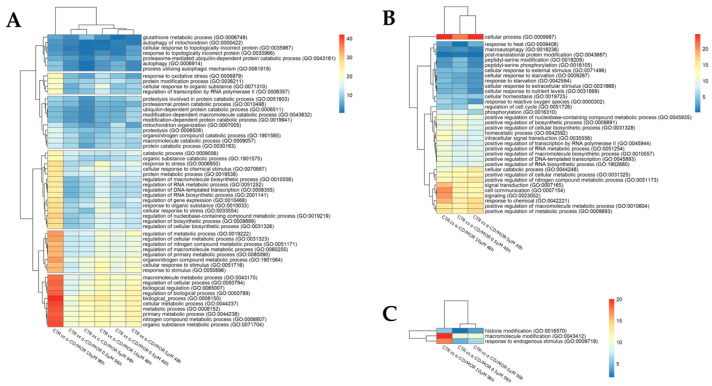
Heatmap of biological processes commonly overrepresented in the different comparisons based on ratio multiplied by total absolute fold change. Frame (**A**) is related to the ontologies commonly overrepresented in all the comparisons. The frames (**B**,**C**) show the ontologies commonly expressed in all doses only at 48 h or 96 h, respectively. The cell filling represents the ratio between the number of DEGs observed in a comparison included in a specific GO and the number of genes included in that ontology term multiplied by the total absolute fold change of the considered DEGs. Red represents groups with a higher score, while blue represents those with lower values. The dendrogram clusters are obtained using a generalized linear model.

The second heatmap, in Figure 4, was obtained by calculating for each group the product of the same ratio previously evaluated, multiplied by the sum of the absolute fold change of the DEGs included in the term. 

This score includes a qualitative information related to the concrete deregulation of the terms. Indeed, even if a few DEGs can be altered in a term, their fold change could be very strong. Conversely, it is not obvious that a term with a huge number of DEGs is a very altered term because the fold change of DEGs in that term could be weak. These heatmaps suggested that the group treated with 10 µM α-CD/MOR showed the highest fold changes.

**Figure 5:**
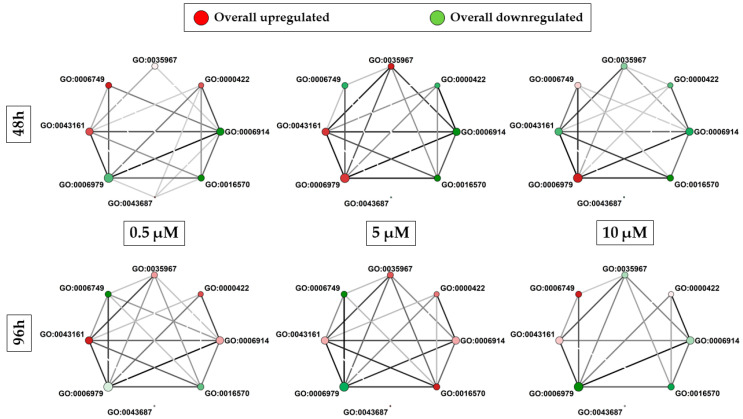
Network of biological processes for each comparison. The size of each node is proportional on a logarithmic scale to the number of DEGs included in the ontology term. The scale color of the nodes from green to red highlights the sum of the fold change values of all DEGs in the term. The different shades of green indicate a downregulation of the term, with darker green that results in a stronger downregulation of the term. Instead, the different shades of red indicate upregulation of the term, with darker red that shows a stronger upregulation. White filling of the nodes indicates a balanced deregulation. The darker the edge that links to nodes, the higher the number of DEGs that are simultaneously deregulated in the two linked terms.

From the heatmaps in Figure 3, we selected the most impacted BPs, and we built for each comparison, so for each dose and time, a representative network, shown in Figure 5, of the GO terms on which we focused our attention. In detail, we linked terms that share DEGs and highlighted an overall deregulation of the terms based on the DEGs that are included. Different DEGs are common between the different BP, indicating that these DEGs take part in various processes, and then the BP are not separate entities, but one influences the others. We observed that the highest edge values were observed in the group treated for 48 h with a concentration of 5 μM and in the group treated for 96 h with a concentration of 10 μM. Specifically, 9 DEGs are shared between “autophagy” (GO:0006914) and “autophagy of mitochondrion” (GO:0000422) in both comparisons. Additionally, GO:0006914 and “response to oxidative stress” (GO:0006979) have nine common DEGs in the group treated for 48 h with the concentration 5 μM. However, the GO:0006979 showed higher and stronger connections for all the comparisons, indicating that all these processes are involved in the oxidative stress response.

### 3.5. Western Blot Analysis 

It is known that Nrf2 also has cytoprotective effects through stimulation of the autophagy process, through which both toxic protein aggregates and dysfunctional organelles can be degraded [33]. Moreover, the BP “autophagy” showed a higher ratio among the common BP. 

**Figure 6:**
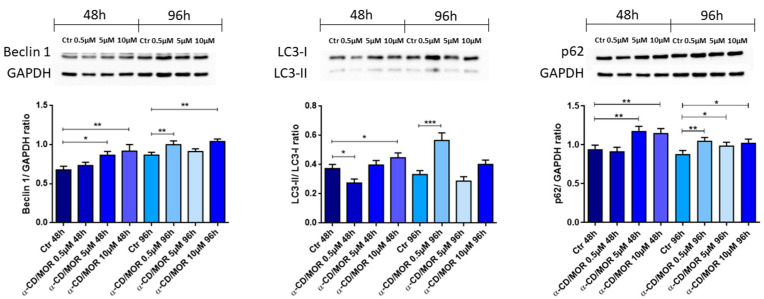
Western blot analysis of Beclin 1, LC3, and p62. Beclin 1 levels increased with the highest concentration both at 48 h and 96 h. LC3-II levels increased with 10 μM α-CD/MOR for 48 h and at 0.5 μM α-CD/MOR for 96 h. The level of p62 protein increased in all groups except in the group treated with 0.5 μM α-CD/MOR for 48 h. * *p* < 0.05; ** *p* < 0.01; *** *p* < 0.001.

In order to evaluate the effect of α-CD/MOR on a cellular self-protection mechanism such as autophagy, the protein expression levels of Beclin 1, LC3-II, and p62 were evaluated, as reported in Figure 6. 

Beclin 1 levels increased after 48 h with the concentrations of 5 and 10 μΜ α-CD/MOR. An increase was also found after 96 h in the groups treated with 0.5 and 10 μΜ α-CD/MOR compared to the control.

LC3-II decreased only at the concentration of 0.5 α-CD/MOR for 48 h. On the contrary, the treatment with concentrations of 10 μΜ for 48 h and 0.5 μΜ for 96 h increased its levels.

Although p62 levels were similar to control in cells treated for 48 h with α-CD/MOR 0.5 μΜ, the data in Figure 6 show the ability of α-CD/MOR to significantly increase the levels of p62 protein in the other groups and this effect may be a consequence of Nrf2 activation.

**Figure 7:**
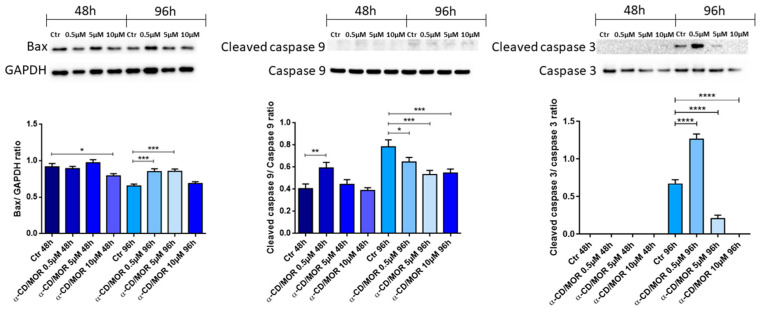
Western blot analysis of apoptotic proteins. Bax levels increased at 96 h with concentrations of 0.5 and 5 μM α-CD/MOR. Cleaved caspase 9 levels decreased in the groups treated for 96 h. Cleaved Caspase 3 was present only in the groups treated for 96 h, except with 10 μM α-CD/MOR. In particular, the group treated with α-CD/MOR at a concentration of 0.5 μM showed the highest levels. * *p* < 0.05; ** *p* < 0.01; *** *p* < 0.001; **** *p* < 0.0001.

Furthermore, we investigated the effect of α-CD/MOR on the apoptotic pathway by assessing the protein expression levels of Bax, cleaved caspase 9, and cleaved caspase-3, reported in Figure 7. The results showed that Bax levels decreased after 48 h of treatment at a concentration of 10 μM α-CD/MOR, while their levels increased at 96 h with the concentrations of 0.5 and 5 μM α-CD/MOR. Cleaved caspase 9 levels increased in the group treated with a concentration of 0.5 μM α-CD/MOR for 48 h. Interestingly, its levels decreased in cells treated for 96 h with all the concentrations. After 48 h of exposure to α-CD/MOR, although the protein levels of caspase-3 were detectable, it was not possible to observe the protein expression of cleaved caspase-3. However, the 96 h treatment led to an increase in the active form. In particular, cleaved caspase-3 protein levels were increased with α-CD/MOR 0.5 μM. In the group treated with α-CD/MOR 5 μM, cleaved caspase 3 was present but reduced compared to the control group. Interestingly, cleaved caspase-3 was not detectable with α-CD/MOR 10 μM after 96 h of exposure.

## 4. Discussion

### 4.1. Neuroprotective Capacity of MOR

MOR has shown several health promoting effects and neuroprotective capacities. In a previous study, we already demonstrated that α-CD/MOR exerted neuroprotective effects in an in vitro model of AD. Specifically, it downregulated the genes encoding for proteins that participate in the processes of senescence, autophagy, and mitophagy. Moreover, α-CD/MOR modulated axon guidance, inducing neuronal remodeling [12]. However, there is not a lot of data regarding the effects of long-term supplementation with MOR. Cell viability results showed only a slight decrease in cells treated with a concentration of 0.5 μM α-CD/MOR for 96 h. Then, in this study, we tried to elucidate the effects of a prolonged supplementation with α-CD/MOR in differentiated NSC-34 motor neurons. With this aim, we performed a transcriptomic analysis of differentiated NSC-34 cells treated with the concentrations of 0.5, 5, and 10 µM for 48 h and 96 h. 

### 4.2. MOR Can Activate Nrf2

ITCs are well-known for their antioxidant properties, and their capacity to activate the transcription factor Nrf2 has already been demonstrated for some ITCs, including Phenethyl Isothiocyanate and SFN [34,35,36]. *Moringa oleifera* extract was reported to be able to activate Nrf2 [9] and also isolated MOR activated Nrf2 in different experimental models [11,13]. Moreover, Nrf2 activation seemed promising for the treatment of some neurodegenerative diseases, such as ALS. Indeed, motor neurons are sensitive to oxidative stress, which can trigger their degeneration. 

In this study, at first, we evaluated if α-CD/MOR was able to activate Nrf2 both at transcriptional and protein levels. We found that the gene *Nfe2l2* was upregulated after 48 h only at a concentration of 10 µM. Instead, after 96 h of treatment, all the concentrations tested were able to significantly increase *Nfe2l2* transcriptional levels. In line with these results, western blot analysis showed an increase in cytoplasmic Nrf2 levels with all the concentrations tested after 96 h of treatment. Moreover, nuclear protein levels of Nrf2 increased at a concentration of 0.5 µM and mainly at 10 µM at 96 h. 

### 4.3. BP Analysis of Nrf2 Interactors

We selected Nrf2 interactors using STRING and evaluated which of them were DEGs in the various comparisons and their overrepresented BP of GO. In particular, we selected the BP common to all groups to evaluate α-CD/MOR effects that were not dependent on time or dose. The classification of the BP evidenced the presence of different clusters based on similar trends in the ratio of the groups. A cluster is represented by only one biological process, which is “glutathione metabolism”. Another big cluster is formed by BP with a more similar ratio and contains various BP related to metabolic processes, specifically transcription, DNA, RNA, and protein metabolism. Interestingly, the BP “response to oxidative stress” is also part of this cluster. In another cluster, “autophagy” and BP related to catabolic processes were represented. Ubiquitin-dependent and proteasome-mediated protein catabolic processes belonged to another cluster. Another cluster included BP related to topologically incorrect proteins. The last cluster included BP relative to mitochondria and catabolic processes. It is interesting to note that in most cases, the groups treated with 10 µM α-CD/MOR at both timepoints showed a higher ratio. However, for the cluster including catabolic processes and autophagy, the groups treated for 48 h with 0.5 and 5 µM had a similar ratio. Of note, all these processes are also involved in ALS pathogenesis, so it is of particular interest the finding that α-CD/MOR may modulate them. 

### 4.4. α-CD/MOR Effects on GSH

GSH has a crucial role in the antioxidant response and in the preservation of neuronal redox homeostasis. Indeed, GSH reduction is found in the brains of patients affected by neurodegenerative diseases [37,38]. GSH synthesis is tightly regulated, and it depends on the availability of cysteine and the activity of the rate-limiting enzyme glutamate cysteine ligase (GCL). The *Slc7a11* gene encodes a cystine/glutamate antiporter Xct, and it provides the cell with the cystine needed for GSH synthesis. GCL is formed by a catalytic subunit that is encoded by the gene *Gclc* and a modifier subunit encoded by the gene *Gclm*. The second enzyme involved in GSH synthesis is GSH synthetase, encoded by the *Gss* gene. In our study, we found that only the group treated for 96 h with 10 µM α-CD/MOR showed the upregulation of all these genes needed for GSH synthesis, while in the group treated with 10 µM for 48 h, only the gene *Gss* was not a DEG. However, it is interesting to notice that all the groups showed upregulation of the *Gpx4* gene. GPX4 has a main role in cystine metabolism and in counteracting lipid peroxidation [39,40]. It is known that Gpx4 is a potent inhibitor of ferroptosis thanks to its capacity to decrease peroxidized membrane lipids. GPX4 detects and reduces peroxidation signals using antioxidant substrates, specifically the GSH system [41]. 

### 4.5. α-CD/MOR Effects on Autophagy

Nrf2, through its interactors, regulates different cellular processes, including autophagy and proteostasis [42]. Autophagy represents a quality control process able to eliminate and recycle cellular components, such as protein aggregates and injured organelles. Nrf2 can also influence autophagy. Specifically, p62 links Nrf2 with autophagy [43]. p62 is a selective autophagy-related protein that contributes to cellular homeostasis by performing two main functions at the physiological level: the effective autophagic degradation of ubiquitinated proteins and the antioxidant stress response [44]. In particular, p62 promotes degradation of Keap1, maintaining high levels of active Nrf2 [45,46,47]. Moreover, Nrf2 activates p62 to maintain redox homeostasis through a positive feedback mechanism [48]. Our results showed the upregulation of p62 encoded by the gene *Sqstm1* in all groups except 10 µM α-CD/MOR for 96 h. Western blot analysis also confirmed the p62 increase. The increase of p62 in the group treated with 10 µM α-CD/MOR for 96 h may be due to a slowdown of autophagy. The Beclin 1 protein plays a crucial role in initiating autophagy. At the physiological level, Beclin 1 is essential for maintaining neuronal health through the process of autophagy [49]. In our analysis, the *Becn1* gene was found to be upregulated after 48 h of treatment. On the contrary, its expression was reduced after 96 h. In line with transcriptomic data, Beclin 1 protein levels increased after 48 h with the concentrations of 5 and 10 μM α-CD/MOR. However, Beclin 1 protein levels were not reduced after 96, indicating that autophagy may be slowed down, but not suppressed. 

LC3 plays a main role in autophagosome elongation and formation. LC3-II represents the conjugated form of cytosolic LC3-I with phosphatidylethanolamine on the surface of autophagosomes [50]. LC3-II protein levels increased after 48 h with the concentration of 10 μM α-CD/MOR, suggesting that autophagy is activated, in line with the other transcriptomic and western blot results. At 96 h, LC3-II also increased after 96 h at the concentration of 0.5 μM α-CD/MOR. The simultaneous increase in both p62 and LC3 is not surprising. Indeed, we mentioned the positive feedback mechanism existing between Nrf2 and p62 that led to an increase in p62 levels. Then, in this experimental model, p62 is enough to mediate both autophagy and Nrf2 activation.

Also, some ATG protein-encoding genes (*Atg5* and *Atg7*) were upregulated in some groups after 48 h of treatment. The data suggested that autophagy is more activated at 48 h of treatment, while it is reduced or more similar to control after 96 h of treatment. Indeed, excessive autophagy may be detrimental for neurons [51,52,53,54]. 

### 4.6. α-CD/MOR Effects on Proteostasis

As said before, different BP common to all groups are involved in proteostasis, such as GO:0043161 “proteasome-mediated ubiquitin-dependent protein catabolic process” and GO:0035967 “cellular response to topologically incorrect protein”. Proteostasis indicates the homeostatic regulation of proteins, from their synthesis and folding to their trafficking and elimination. In particular, unfolded or misfolded proteins represent a feature of different diseases, including neurodegenerative diseases. Their accumulation can lead to ER stress, triggering the Unfolded protein response (UPR) through the activation of the three signaling arms regulated by IRE1, PERK, and ATF6. Interestingly, the genes *Ern1*, *Eif2ak3,* and *Atf6*, encoding for IRE1, PERK, and ATF6, respectively, were downregulated in cells treated for 96 h with 10 µM α-CD/MOR. Also, *Xbp1*, a mediator acting downstream on IRE1, was downregulated. These data indicated a reduction in UPR. Interestingly, *Hspa5* which encodes for Bip and is also known as GRP78, and the gene *Ddit3,* which encodes for CHOP, were downregulated by the treatment with a concentration of 10 µM α-CD/MOR for 96 h. GRP78 helped the unfolded proteins to fold or induce their degradation [55], while CHOP plays a fundamental role in ER stress-induced apoptosis [56]. 

Nrf2 can also regulate the genes encoding for subunits of the 19S and 20S proteasomes [42]. Interestingly, the concentration of 10 µM at both time points showed the upregulation of the core subunits of the 20S proteasome *Psmb5* and *Psma5*. Instead, the other concentrations showed only upregulation of *Psma5* after 48 h of incubation. Indeed, at 96 h, there is mainly a downregulation of proteasome subunits, except at 10 µM. 

### 4.7. α-CD/MOR Effects on the Other Significant BP and Their Network

Moreover, Nrf2 is also able to modulate mitochondrial dynamics [57,58], as also shown by the modulated common BP regarding mitochondria. In particular, Nrf2 maintains mitochondrial homeostasis, regulating both fusion, fission, and mitophagy [58]. 

Looking for the BP common to all the comparisons at 48 h, it is evident that all showed a similar ratio, except for “post-translational protein modification”. This BP includes exclusively the genes *Atg5* and *Atg7,* and the group treated with 0.5 µM is the only one to express them all. At 96 h, only 3 BP are common between the 3 groups, with the BP “Histone modification” showing the highest ratio. It is reported that histone deacetylases may also play a role in the degeneration of motor neurons [59]. Different ITCs were reported to induce epigenetic change by modulating histone deacetylases [34,60]. However, it is possible to observe that fewer differences were present in common BP at 48 h than at 96 h. This suggests that the different α-CD/MOR concentrations change the expression of a similar number of DEGs for the common BP. 

We also considered the magnitude of fold change for the common BP in the different groups. In this context, it is evident that the group treated with 10 µM α-CD/MOR for 96 h showed the highest fold changes. In particular, differences in the score regarding fold change are evident for the second cluster in Figure 4, in which the GO “response to oxidative stress” is present. Also looking at the common BP for 48 h and 96 h, the results showed that the concentration of 10 µM α-CD/MOR showed the highest magnitude regarding fold changes. 

The network of the most impacted BP evidenced that the different BP share some common DEGs, indicating that all BP are associated to mediate α-CD/MOR effects. In particular, the group treated with 10 µM α-CD/MOR for 96 h presented the highest edge value. 

### 4.8. α-CD/MOR Effects on Apoptosis

We also evaluated the induction of apoptosis by analyzing Bax, cleaved caspase 9, and cleaved caspase 3 levels. Western blot evidenced that Bax was reduced at a concentration of 10 µM α-CD/MOR for 48 h, while it slightly increased at 0.5 and 5 µM α-CD/MOR at 96 h. Cleaved caspase 9 levels decreased after 96 h of treatment. Cleaved caspase 3 was visible only after 96 h of incubation, also in control, probably due to the prolonged time in culture. Of note, the group treated with 0.5 µM α-CD/MOR showed the highest level of caspase 3 activation. Interestingly, the group treated with 10 µM α-CD/MOR showed no cleaved caspase 3. Then, the results indicated that the group treated with the highest concentration for 96 h, showed the lowest levels of apoptotic markers.

### 4.9. Future Perspective

In this study, we evaluated the effects associated with prolonged administration of α-CD/MOR. Indeed, this treatment is intended to be used as an integrative therapy with the aim of disease prevention. Then, it should be administered for a long period of time to healthy subjects. The results obtained suggested that prolonged administration is not associated with toxic effects; instead, it induces positive effects on motor neurons. Then, our future prospective is to evaluate its neuroprotective effects after prolonged administration in oxidative stress-induced models.

To our knowledge, this is the first study to evaluate the effects of prolonged treatment with isolated MOR. Previously, a study evaluated the effects of long-term consumption of *Moringa oleifera* leaves in rats, demonstrating protection against oxidative stress and hippocampal degeneration and improvements in neurocognition [61].

## 5. Conclusions

In this study, we found that the prolonged treatment of differentiated NSC-34 motor neurons with α-CD/MOR was able to activate Nrf2 and its interactors. In particular, BP analysis evidenced that α-CD/MOR was able to induce an antioxidant gene expression profile modulating glutathione synthesis genes, and the effect was more evident with the highest dose at 96 h. Moreover, autophagy and proteostasis BP were modulated, which participate in the antioxidant response. The effects were a reduction of ER stress markers and an increased expression of proteasome subunits, suggesting a reduction of proteotoxicity. These data suggested that α-CD/MOR exerted health-promoting effects, at least in part mediated by the Nrf2 antioxidant response. Then, these results suggested that α-CD/MOR modulates processes involved in motor neuron cell death and can be a valuable integrative therapy against motor neuron degeneration.

## Data Availability

The data presented in this study are openly available in the NCBI Sequence Read Archive at BioProject, accession number PRJNA1117421.

## Supplementary Materials

The following supporting information can be downloaded at: https://www.mdpi.com/article/10.3390/antiox13070813/s1. Figure S1: Uncropped blots for cytoplasmic Nrf2 and GAPDH, and for nuclear Nrf2 and Lamin B1; Figure S2: Uncropped blots for Beclin 1 and GAPDH; Figure S3: Uncropped blot for LC3; Figure S4: Uncropped blots for p62 and GAPDH; Figure S5: Uncropped blots for Bax and GAPDH; Figure S6: Uncropped blots for cleaved caspase 9 and caspase 9; Figure S7: Uncropped blots for cleaved caspase 3 and caspase 3; Table S1: DEGs in CTR vs. 0.5 µM α-CD/MOR 48 h; Table S2: DEGs in CTR vs. 5 µM α-CD/MOR 48 h; Table S3: DEGs in CTR vs. 10 µM α-CD/MOR 48 h; Table S4: DEGs in CTR vs. 0.5 µM α-CD/MOR 96 h; Table S5: DEGs in CTR vs. 5 µM α-CD/MOR 96 h; Table S6: DEGs in CTR vs. 10 µM α-CD/MOR 96 h; Table S7: Nrf2 interactors in STRING database; Table S8: Overrepresented BP terms in CTR vs. 0.5 µM α-CD/MOR 48 h; Table S9: Overrepresented BP terms in CTR vs. 5 µM α-CD/MOR 48 h; Table S10: Overrepresented BP terms in CTR vs. 10 µM α-CD/MOR 48 h; Table S11: Overrepresented BP terms in CTR vs. 0.5 µM α-CD/MOR 96 h; Table S12: Overrepresented BP terms in CTR vs. 5 µM α-CD/MOR 96 h; Table S13: Overrepresented BP terms in CTR vs. 10 µM α-CD/MOR 96 h; Table S14: Overrepresented BP terms shared by all comparisons; Table S15: Overrepresented BP terms shared by comparisons at 48 h; Table S16: Overrepresented BP terms shared by comparisons at 96 h.
