# Supplementary material for: α-Cyclodextrin/Moringin Induces an Antioxidant Transcriptional Response Activating Nrf2 in Differentiated NSC-34 Motor Neurons
Source: Antioxidants (Basel). 2024 Jul 6;13(7):813. doi: 10.3390/antiox13070813 (PMC11274022; doi:10.3390/antiox13070813)
Supplement: Supplementary file 1 [file antioxidants-13-00813-s001.zip › Captions of Supplementary Materials.pdf]

Figure S1. Uncropped blots for cytoplasmic Nrf2 and GAPDH, and for nuclear Nrf2 and Lamin B1.
Figure S2. Uncropped blots for Beclin 1 and GAPDH.
Figure S3. Uncropped blot for LC3.
Figure S4. Uncropped blots for p62 and GAPDH.
Figure S5. Uncropped blots for Bax and GAPDH.
Figure S6. Uncropped blots for cleaved caspase 9 and caspase 9.
Figure S7. Uncropped blots for cleaved caspase 3 and caspase 3.
Table S1. DEGs in CTR vs. 0.5 μM α-CD/MOR 48 h.
Table S2. DEGs in CTR vs. 5 μM α-CD/MOR 48 h.
Table S3. DEGs in CTR vs. 10 μM α-CD/MOR 48 h.
Table S4. DEGs in CTR vs. 0.5 μM α-CD/MOR 96 h.
Table S5. DEGs in CTR vs. 5 μM α-CD/MOR 96 h.
Table S6. DEGs in CTR vs. 10 μM α-CD/MOR 96 h.
Table S7. Nrf2 interactors in STRING database.
Table S8. Overrepresented BP terms in CTR vs. CTR vs. 0.5 μM α-CD/MOR 48 h.
Table S9. Overrepresented BP terms in CTR vs. CTR vs. 5 μM α-CD/MOR 48 h.
Table S10. Overrepresented BP terms in CTR vs. CTR vs. 10 μM α-CD/MOR 48 h.
Table S11. Overrepresented BP terms in CTR vs. CTR vs. 0.5 μM α-CD/MOR 96 h.
Table S12. Overrepresented BP terms in CTR vs. CTR vs. 5 μM α-CD/MOR 96 h.
Table S13. Overrepresented BP terms in CTR vs. CTR vs. 10 μM α-CD/MOR 96 h.
Table S14. Overrepresented BP terms shared by all comparisons.
Table S15. Overrepresented BP terms shared by comparisons at 48 h.
Table S16. Overrepresented BP terms shared by comparisons at 96 h.
