# Supplementary figures and images for: α-Cyclodextrin/Moringin Induces an Antioxidant Transcriptional Response Activating Nrf2 in Differentiated NSC-34 Motor Neurons
Source: Antioxidants (Basel). 2024 Jul 6;13(7):813. doi: 10.3390/antiox13070813 (PMC11274022; doi:10.3390/antiox13070813)

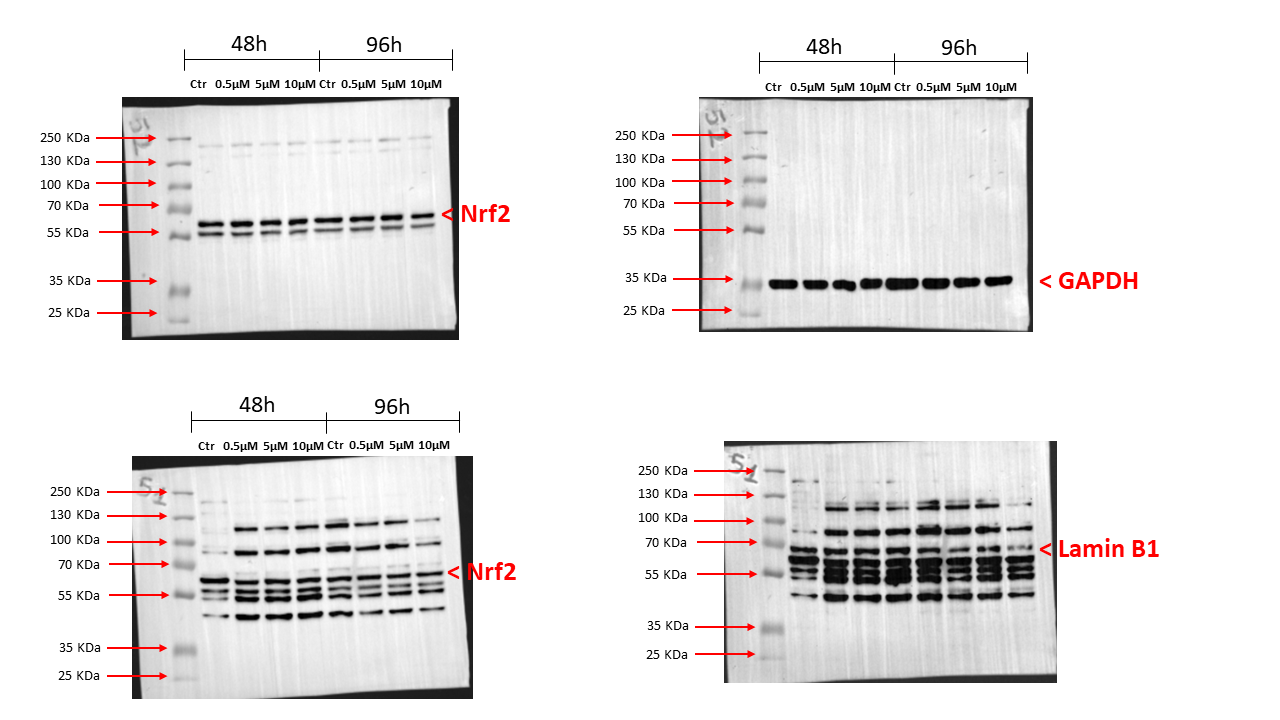

Supplement: Supplementary file 1 [file antioxidants-13-00813-s001.zip › Figure S1.TIF]

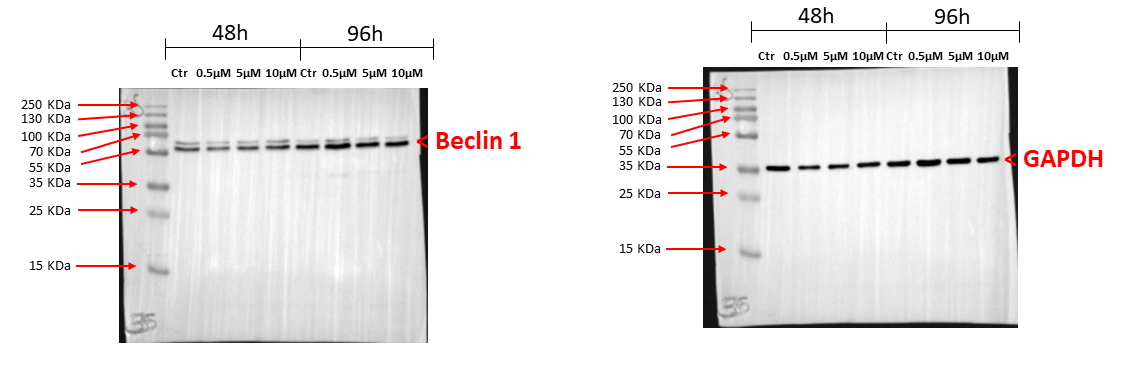

Supplement: Supplementary file 1 [file antioxidants-13-00813-s001.zip › Figure S2.TIF]

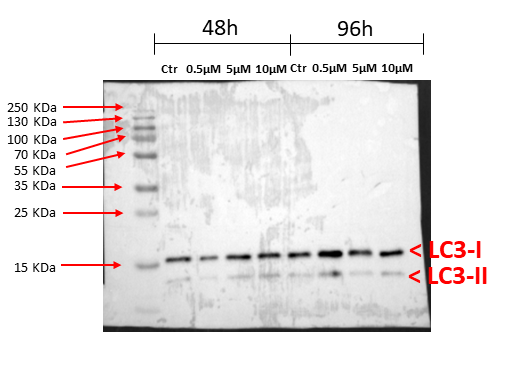

Supplement: Supplementary file 1 [file antioxidants-13-00813-s001.zip › Figure S3.TIF]

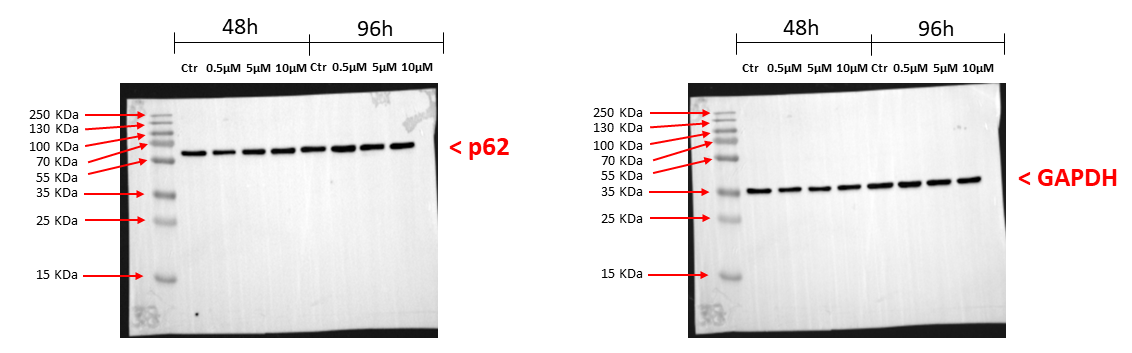

Supplement: Supplementary file 1 [file antioxidants-13-00813-s001.zip › Figure S4.TIF]

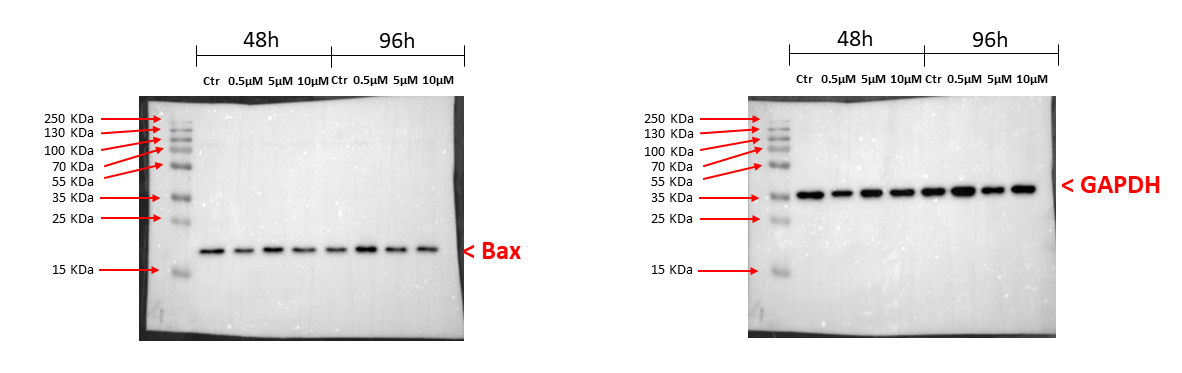

Supplement: Supplementary file 1 [file antioxidants-13-00813-s001.zip › Figure S5.TIF]

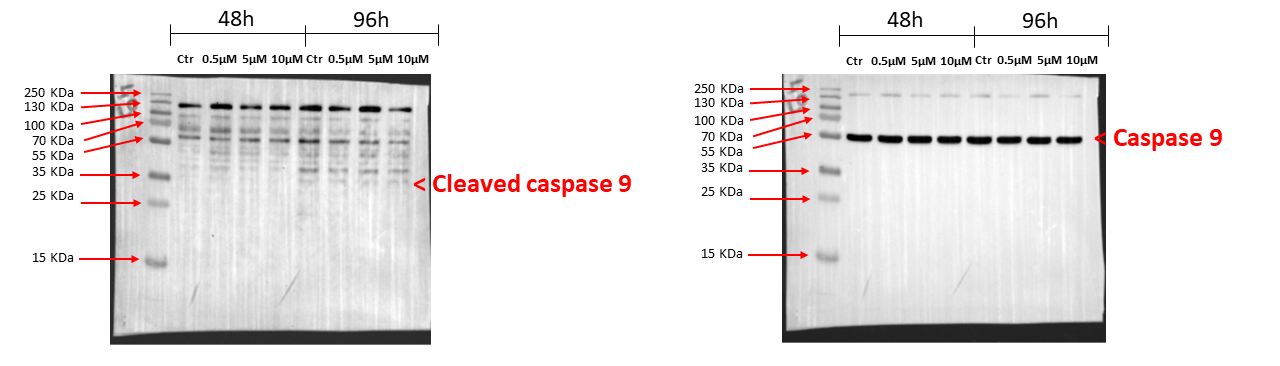

Supplement: Supplementary file 1 [file antioxidants-13-00813-s001.zip › Figure S6.TIF]

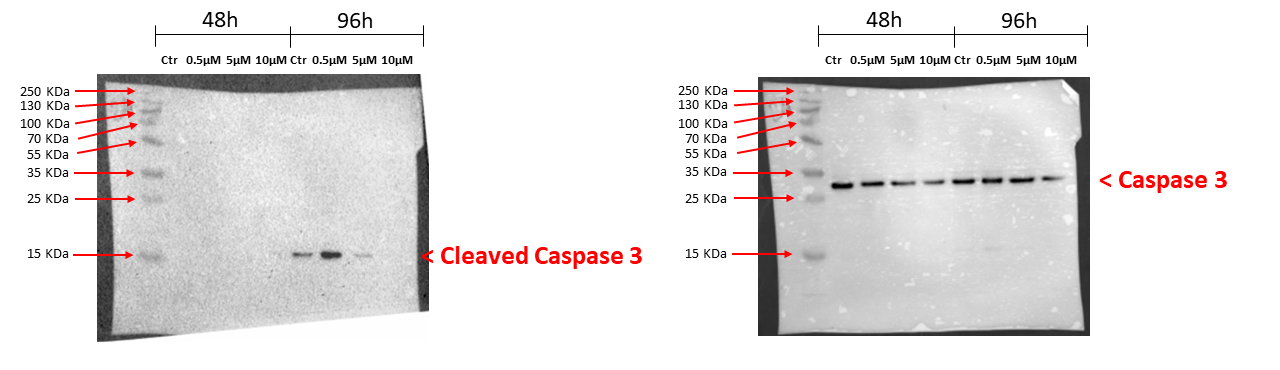

Supplement: Supplementary file 1 [file antioxidants-13-00813-s001.zip › Figure S7.TIF]
